# Scraps

**Published:** 1886-06

**Authors:** 


### SCRAPS

PICKED UP BY THE ASSISTANT - EDITOR.

---

**The Wrong Place.**—"It's no use to feel av me wrist, doctor," said Pat, when the physician began taking his pulse: "the pain is not there, sir; it's in me head entoirely."

**Things one would rather have left unsaid.**—Patient: "I am so sorry, doctor, I was obliged to send for you so late in the day, and to bring you this long way on purpose to see *me*." Doctor: "Don't name it. I had another person to see close by, and I can kill two birds with one stone!"

**Another Hermaphrodite.**—Among the replies to an advertisement of a musical committee for "a candidate as organist, music-teacher, &c." was the following one: "Gentlemen,—I noticed your advertisement for an organist and music-teacher, either lady or gentleman. Having been both for several years, I offer you my services."

**Scientific Enquiry.**—At a local medical meeting a country child of tender years having an anomalous skin-eruption was shown. There was some suspicion that it had a specific origin. An enquirer, more enthusiastic than discriminating, asked the child: "Has your mother ever had any mis-*carriages?*" "No, sir," she said; "mother has always took round the vegetables in a *donkey cart*."

**Was he the Poet's Doctor?**—The *Allgemeine Wiener Medicinische Zeitung* announces that a grave-stone exists in the churchyard of Fredericksburg bearing the following inscription: "Here lies Edward Heldon, a medical and surgical practitioner, the friend and companion of William Shakespeare, of Avon. He died after a short illness in the year of our Lord, 1618, in the 70th year of his age."—*The Medical and Surgical Reporter*, Philadelphia.

**Cheltenham Waters.**—A correspondent in one of the Society papers says: "There can be no doubt but that Cheltenham is looking up as a health resort. In 1885 some fifty-two gallons of the Montpellier Spa waters were consumed. Already this year over a hundred gallons have been taken. I am not surprised at this. The waters are equal to those obtained at any foreign spring. Cheltenham is much more easily reached than any of the continental health-giving towns, and in addition is a very pleasant and pretty place."

**Physiology in Elementary Schools.**—To the question, "Describe the process of digestion," one of the children "presented" in physiology replied: "Food is digested by the action of the lungs. Digestion is brought on by the lungs having something the matter with them. The food then passes through your windpipe to the pores, and thus passes off your body by evaporation through a lot of little holes in your skin called capillaries. The food is nourished in the stomach. If you were to eat anything hard you would not be able to digest it, and the consequence would be you would have indigestion. The gall-bladder throws off juice from the food which passes through it. We call the kidneys the bread-basket, because it is where all the bread goes to. They lay up concealed by the heart."

**Quack Medicines.**—Dr. John Lindsay, in an address on "The Relation of Faith to Medical Treatment," reported in the *Glasgow Medical Journal*

says: "About a century ago an ignorant, vulgar woman, Joanna Stephens got £5,000 from the government of the day for revealing her medicines, which had worked wonders on dukes, duchesses, bishops, and thousands of all sorts. Before giving the £5,000—a very big sum in those days—government, in its wisdom, appointed a commission, which reported favourably as to the utility, efficacy, and dissolving power of the stuffs. They included a powder, a decoction, and pills, and were made of snails, egg-shells, hips and haws, carrot-seeds, soap, &c. This shows the very great value of a government commission."

**Farinaceous Diet.**—In reference to the statement that chemical analysis of pop-corn shows it to contain more albuminoids than most of the other cereals, and that in certain parts of the West it is extensiveiy used as a regular article of food, the *Medical Record* (New York) says that our Pilgrim Fathers made some personal experiments with pop-corn; the result being that they started a Day of Thanksgiving for not having to live on it any longer. Whereupon the *American Practitioner and News* adds: "In the light of the above chemical revelation, the logical fitness and poetic beauty of the following old rhymes are manifest:

'And there they sat a-popping corn,
John Styles and Susan Cutter;
John was as strong as any ox,
And Susan fat as butter.'

That those Calvinistic ascetics known as the Pilgrim Fathers should cloy upon so rich a diet is no matter for surprise when one notes the fondness of their descendants for dry cod-fish, beans, and starved mackerel."

**Town-resurrectioning sixty years ago.**—The time chosen in the dark winter nights was from six to eight o'clock, at which latter hour the churchyard watch was set, and the city police also commenced their night rounds. A hole was dug down to the coffin only where the head lay—a canvas sheet being stretched around to receive the earth, and to prevent any of it spoiling the smooth uniformity of the grass. The digging was done with short, flat, dagger-shaped implements of wood, to avoid the clicking noise of iron striking stones. On reaching the coffin two broad iron hooks under the lid, pulled forcibly up with a rope, broke off a sufficient portion of the lid to allow the body to be dragged out, and sacking was heaped over the whole to deaden the sound of the cracking wood. The body was stripped of the grave-clothes, which were scrupulously buried again; it was secured in a sack, and the surface of the ground was carefully restored to its original condition,—which was not difficult, as the sod over a fresh-filled grave must always present signs of recent disturbance. The whole process could be completed in an hour, even though the grave might be six feet deep, because the soil was loose, and the digging was done impetuously by frequent relays of active men. Transference over the churchyard wall was easy in a dark evening; and once in the street, the carrier of the sack drew no attention at so early an hour. Dr. Knox, who in his time was the most popular lecturer on anatomy in Edinburgh, fell into blamable carelessness in his transactions with Burke and Hare and others. He must have known that the bodies which were brought to him warm and flexible could never have been buried. Although, in the inquiry into his alleged criminality, he was not found guilty, a strong feeling set in against him. His talent as a lecturer kept his class-room crowded as usual, but only for a few years. The numbers fell off at last. He left Edinburgh; and, after various vicissitudes and successive descents, he sank, before his death in London, to a state not much above destitution. One of his last occupations was that of lecturer, demonstrator, or showman, to a travelling party of Ojibbeway Indians.—*The Life of Sir Robert Christison.*

